# "Two-Step Shish Kebab": A Useful Technique for Reverse Shoulder Arthroplasty in Humeral Surgical Neck Non-unions

**DOI:** 10.7759/cureus.104865

**Published:** 2026-03-08

**Authors:** Antoine Van Ravestyn, Jan Van Oyen, Joris De Vos, Olivier Verborgt

**Affiliations:** 1 Department of Orthopaedics and Traumatology, AZ Oostende, Ostend, BEL; 2 Department of Orthopaedics, Orthopaedic Center Antwerp (ORTHOCA) AZ Monica, Antwerp, BEL; 3 Department of Orthopaedic Surgery and Traumatology, University Hospital of Antwerp, Antwerp, BEL; 4 Faculty of Medicine and Health Sciences, University of Antwerp, Antwerp, BEL

**Keywords:** proximal humerus fracture, reverse shoulder arthroplasty, surgical neck non-union, two-step shish kebab, type 3 fracture sequelae

## Abstract

The treatment of surgical neck non-unions of the proximal humerus presents significant challenges, with reverse shoulder arthroplasty emerging as a treatment option. During this procedure, avoiding tuberosity osteotomies is crucial to optimizing functional outcomes and minimizing complications. We introduce the "two-step shish kebab" method, a useful technique where the metaphyseal and tuberosity segments of the proximal humerus are percutaneously reduced and transfixed with a modified Steinmann pin before performing the deltopectoral approach. This pin is subsequently replaced by the humeral stem of the prosthesis, which is inserted directly through the proximal fragment and into the medullary canal, eliminating the need for tuberosity osteotomy. This technique offers an elegant solution to the technical challenges associated with type 3 fracture sequelae.

## Introduction

Proximal humerus fractures account for 4-5% of all fractures, with a rising incidence worldwide, particularly among females and the elderly [1-3]. Non-union following proximal humerus fractures occurs in approximately 1.1-10% of cases [4]. Disimpacted surgical neck non-unions of the proximal humerus, AO/OTA 11-A2 type fractures (proximal, extra-articular, unifocal, two-part, surgical neck) [5], are classified as type 3 fracture sequelae according to the classification described by Boileau et al. [6].

These non-unions are frequently associated with persistent pain, leading to limited shoulder motion and unsatisfactory functional outcomes. As a result, patients experience significant limitations in activities of daily living [6,7].

Several risk factors for non-union have been identified, including two-part fracture patterns, metaphyseal comminution, surgical neck translation, smoking, patient age, osteoporosis, rheumatoid arthritis, and ipsilateral glenohumeral osteoarthritis [4,8].

The management of surgical neck non-unions of the proximal humerus can be quite challenging. Nonoperative treatment leads to satisfying outcomes in very low-demand elderly patients. Joint-preserving strategies, such as internal fixation with low-profile locking plates, with or without bone grafting, or intramedullary nailing, achieve mixed results and are often disappointing in the elderly population [1]. The use of anatomic total shoulder arthroplasty (aTSA) in this setting is limited by poor outcomes and high complication rates, largely resulting from the need for greater tuberosity osteotomy [6,9]. Reverse total shoulder arthroplasty (rTSA) demonstrates better results. The results, however, remain lower and less predictable than other indications for rTSA.

When rTSA is indicated, it is imperative to restore enough real estate of the proximal humerus in order to preserve the tuberosities but also to recreate the deltoid wrapping effect, which is of utmost importance for the function and stability of the prosthesis. This can be done by either reincorporating the metaphyseal fragment in the reconstruction or resecting the proximal bone and using reverse shoulder-allograft prosthesis composite (APC) [10] or segmental reconstruction systems (SRS) that replace the proximal humerus [11].

Transfixing several segments of bone is called the "shish kebab technique" and is used in several posttraumatic situations where surgeons try to preserve bone stock in comminuted, segmental fractures [12-15]. The concept is to transfix bony segments so that they form one, either temporary or permanent, unit, not unlike the combination of the pieces of meat and the transfixing skewer that together form a shish kebab (an English rendering of Turkish: 'şiş' (sword, skewer) and 'kebap' (roasted meat dish)).

Boileau et al. and Rais et al. have demonstrated that avoiding tuberosity osteotomies - especially of the greater tuberosity - is critical for optimizing functional outcomes after reverse shoulder arthroplasty for proximal humerus non-unions [15,16]. If osteotomized, complications frequently occur, such as difficulty in refixation, loss of refixation and redisplacement, or even complete resorption of the tuberosities. In turn, this leads to an unacceptable rate of dislocations due to the absence of the rotator cuff complex and failure to sufficiently restore humeral length [16].

When reconstructing the metaphyseal and tuberosity part of the proximal humerus with an rTSA, the "shish kebab way," the humeral stem can be used as an intramedullary nail, inserting it straight into and through the proximal humeral fragment and then into the medullary canal, thereby avoiding any tuberosity osteotomy [15]. Moreover, if feasible, it provides a reliable alternative for the more complex reconstructive techniques using APCs or SRSs, as mentioned above.

Aside from the usual subacromial and subdeltoid soft-tissue scarring (even when the fracture was initially treated conservatively), the main problem is the struggle to bring and hold the proximal humeral fragment in the correct position during the aforementioned surgery technique [6,15].

We present the "two-step shish kebab technique" that elegantly deals with the technical difficulties associated with these type 3 fracture sequelae.

## Case presentation

**Figure 1:**
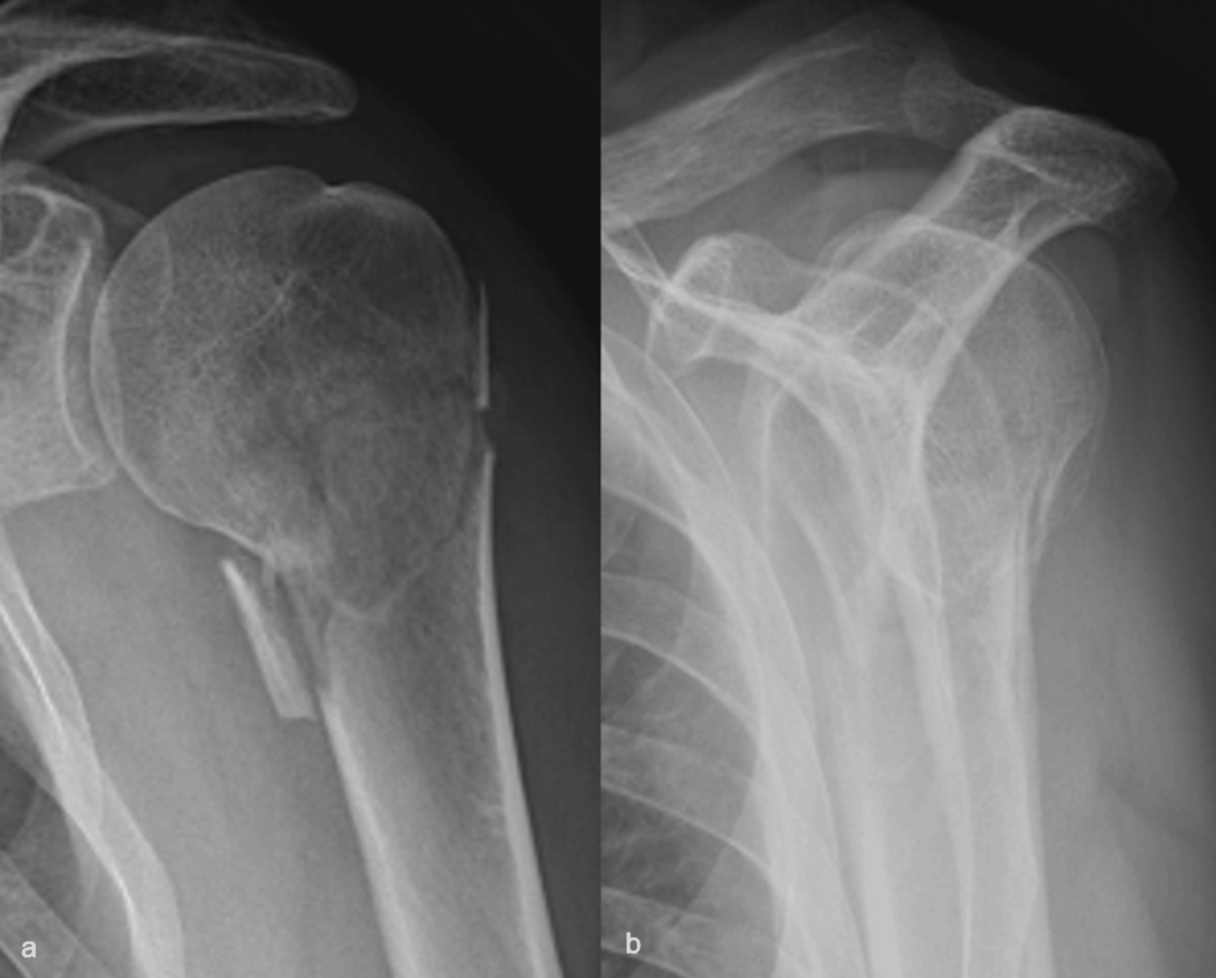
The initial fracture on AP (a) and lateral (b) X-rays: essentially undisplaced with some medial comminution

**Figure 2:**
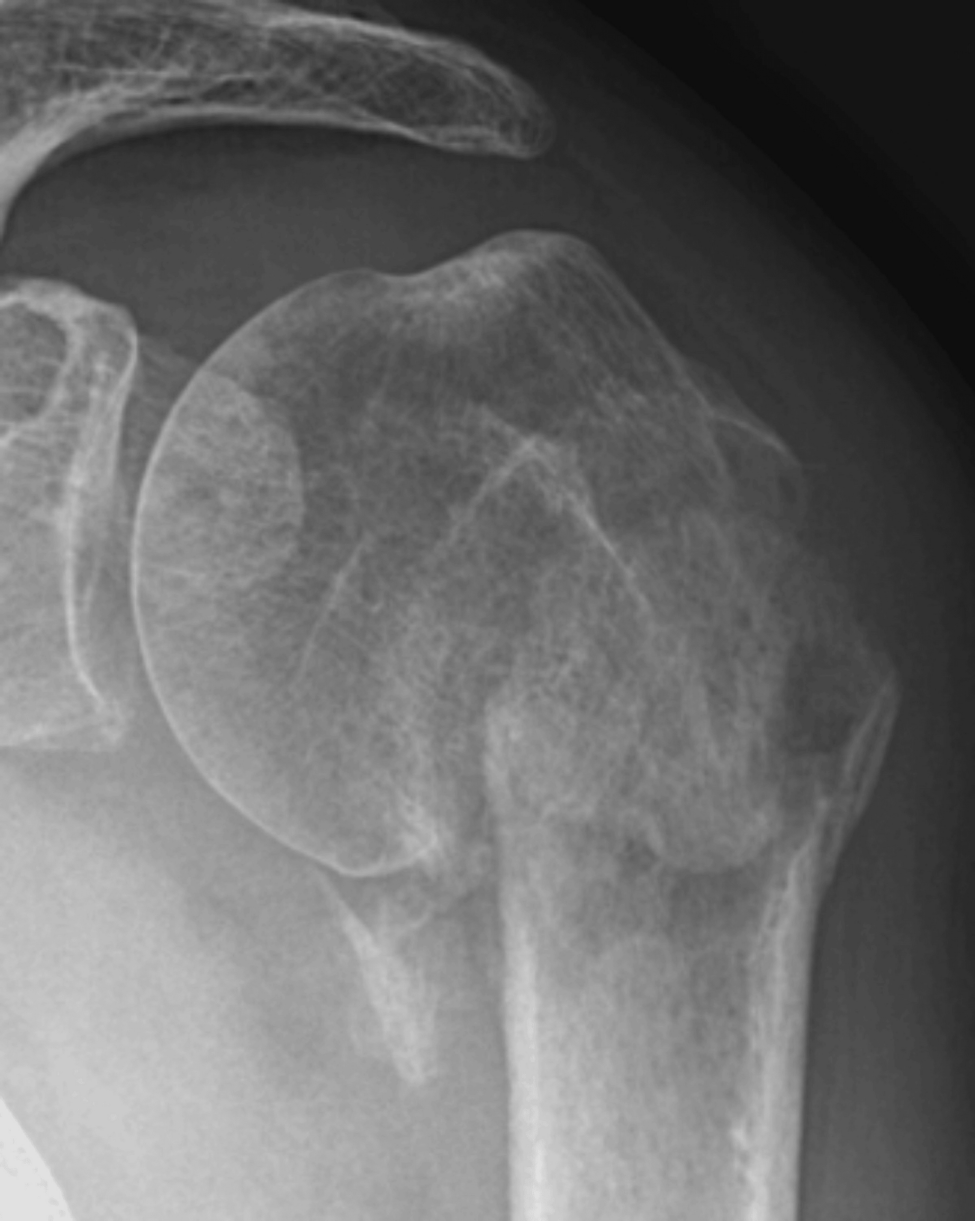
Progressive varus displacement of the fracture fragments during the following months, as seen here after three months

**Figure 3:**
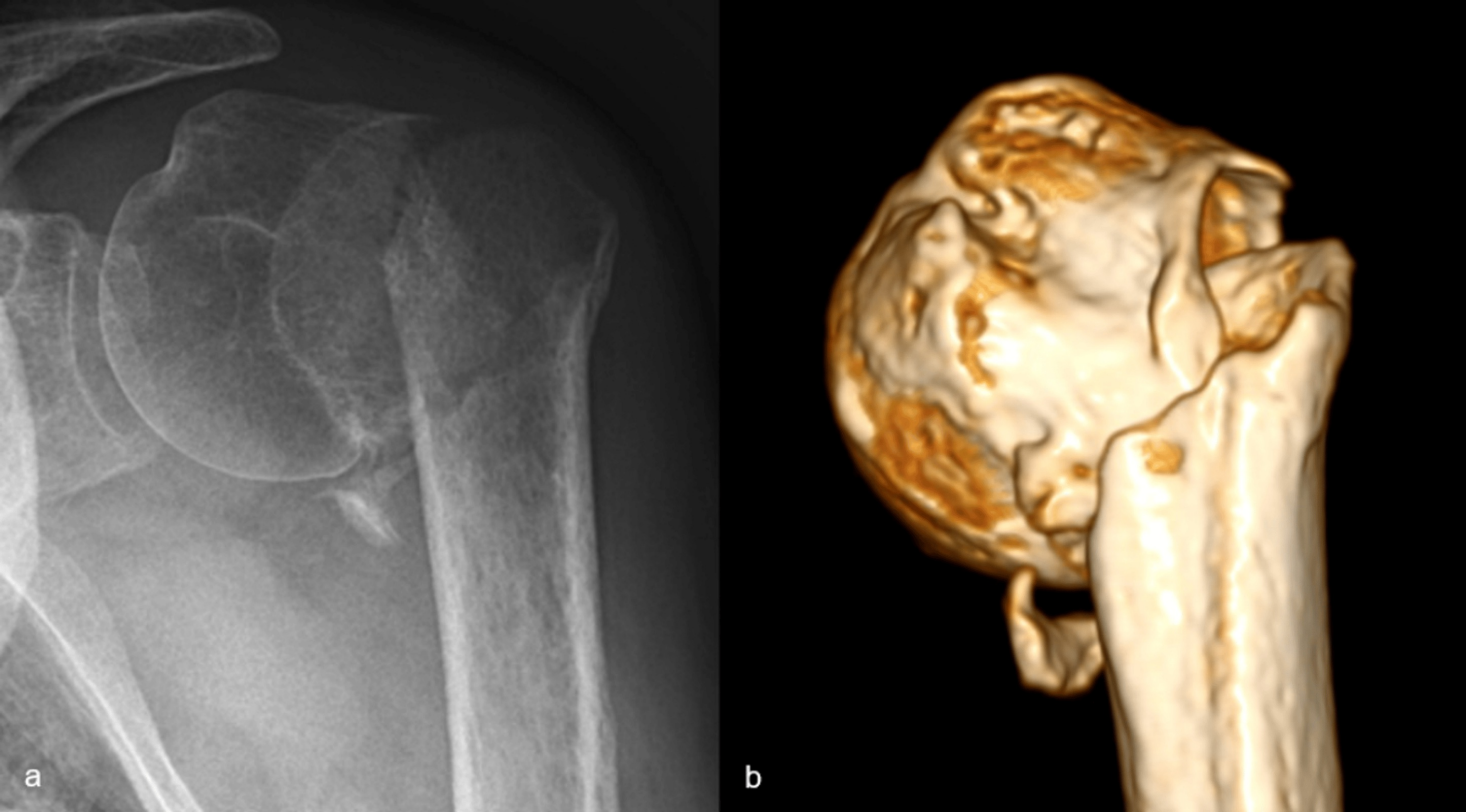
AP X-ray (a) and 3D-CT image reconstruction (b), seven months after the initial trauma: established non-union in about 80° varus position, with excavation of the humeral head, but good cortical bone on both sides CT: computed tomography

A 74-year-old male patient sustained an undisplaced surgical neck fracture of the left proximal humerus (Figure 1). This was treated conservatively with a sling and weekly X-rays for three weeks, after which a home exercise program and formal physiotherapy were started. However, after three months, he presented again with an absence of active elevation and X-rays that showed a progressive varus non-union (Figure 2). Due to comorbidities that had priority at that time, he could not be operated upon until seven months after the initial trauma, at which time he still had no active elevation at all due to an established surgical neck non-union in a varus position of about 80°, with an excavated humeral head, but without a major loss of cortical bone (Figure 3).

Possible options were discussed with the patient. Although the proximal humeral fragment most likely was viable, and osteosynthesis with either a locking nail or locking plate - with or without bone graft - certainly was an option, bony union could not be guaranteed. Due to his age and comorbidities, the patient wanted the operation on his shoulder to be his last, making shoulder arthroplasty the preferred treatment option. Given the poorer functional outcomes of anatomical total shoulder prosthesis for fractures in elderly patients [1,9], he was scheduled for an rTSA. No MRI was performed prior to surgery since it would not change clinical decision-making.

Surgical technique

The patient was positioned in the beach chair position under general anesthesia. Fluoroscopy was installed on the opposite side to obtain both AP and lateral images of the proximal humerus.

**Figure 4:**
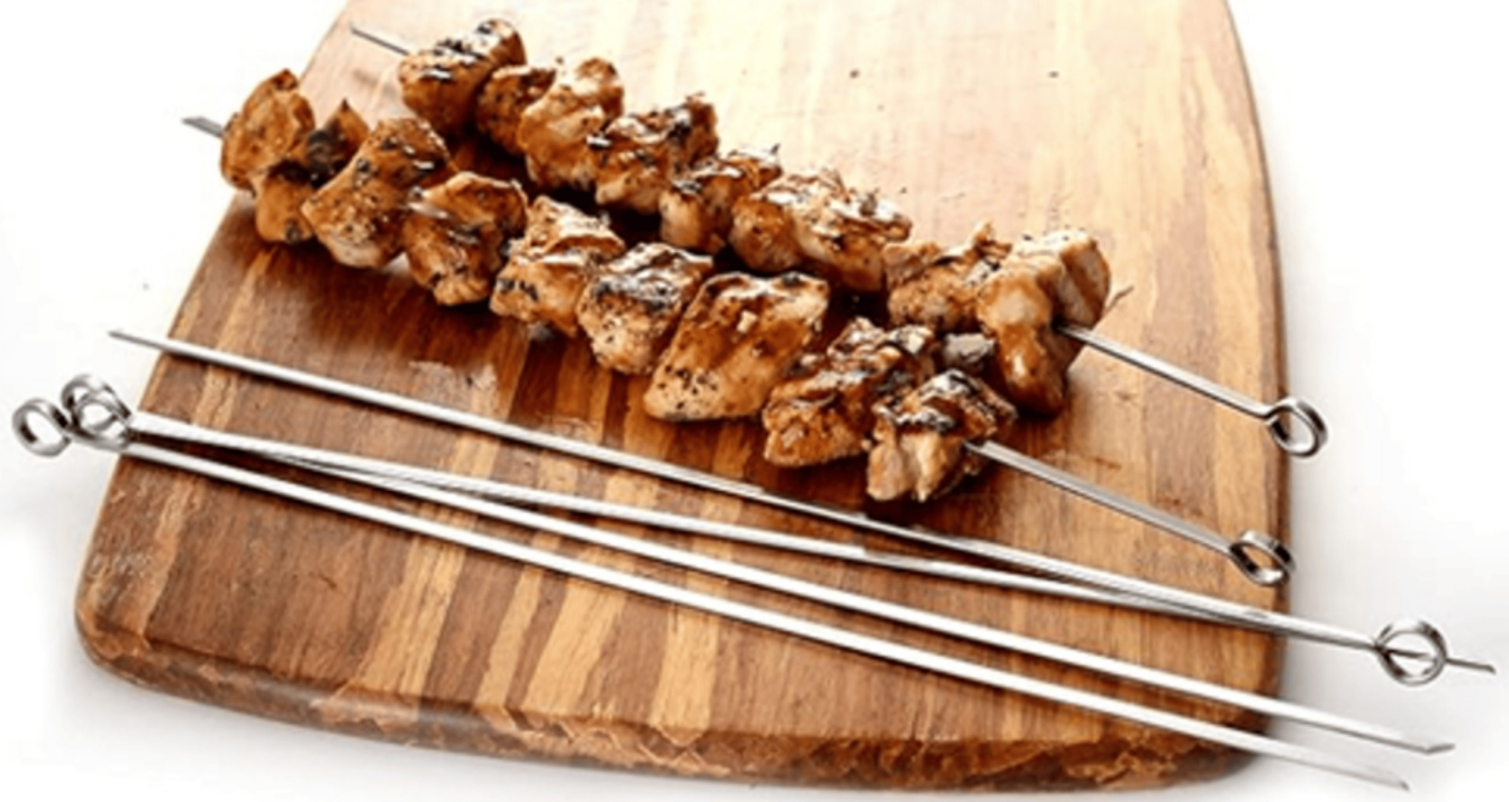
Pieces of meat on skewers: shish kebab "Shish kebab" is a traditional Middle Eastern roasted meat dish in which pieces of meat are threaded onto a skewer. The term originates from the Turkish words "şiş" (sword or skewer) and "kebap" (roasted meat dish). Original image by the authors.

The surgical plan was to execute a "two-step shish kebab technique." First, a modified Steinmann pin was introduced percutaneously through the head and shaft fragments to maintain the initial reduction and facilitate the rest of the procedure. Second, the correctly reduced head and shaft fragment were transfixed with the humeral stem of the reversed shoulder prosthesis while avoiding tuberosity osteotomies. This approach results in sequential axial fixation of both bony fragments, analogous to the alignment of pieces of meat on a skewer in shish kebab, as mentioned previously (Figure 4).

**Figure 5:**
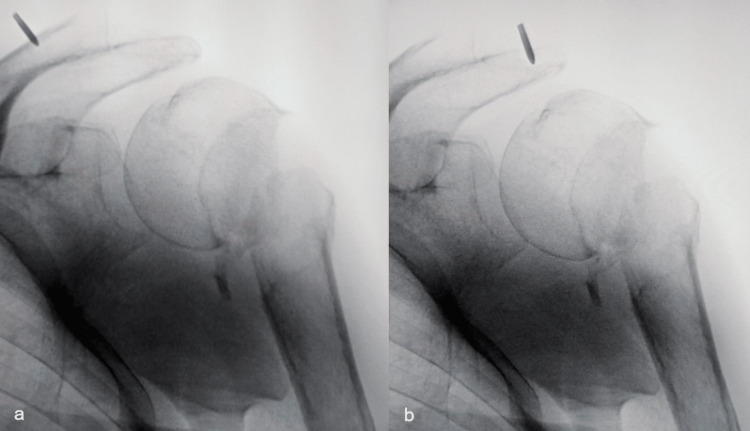
Testing with a ballpoint pen under fluoroscopy shows that access through the Neviaser portal (a) will result in the easiest approach to the most cranial point of the humeral head Access through the Neviaser portal (a) was compared to the anterolateral approach to the proximal humerus (b).

Mobility at the non-union site was confirmed under fluoroscopy. Testing with a ballpoint pen (Figure 5) convinced us that it would be easiest to introduce the Steinmann pin through the Neviaser portal [17], while partially correcting the position of the humeral head with another percutaneously introduced Steinmann pin, all the while aligning the humeral shaft to the position of the humeral head with an arm positioner.

**Figure 6:**
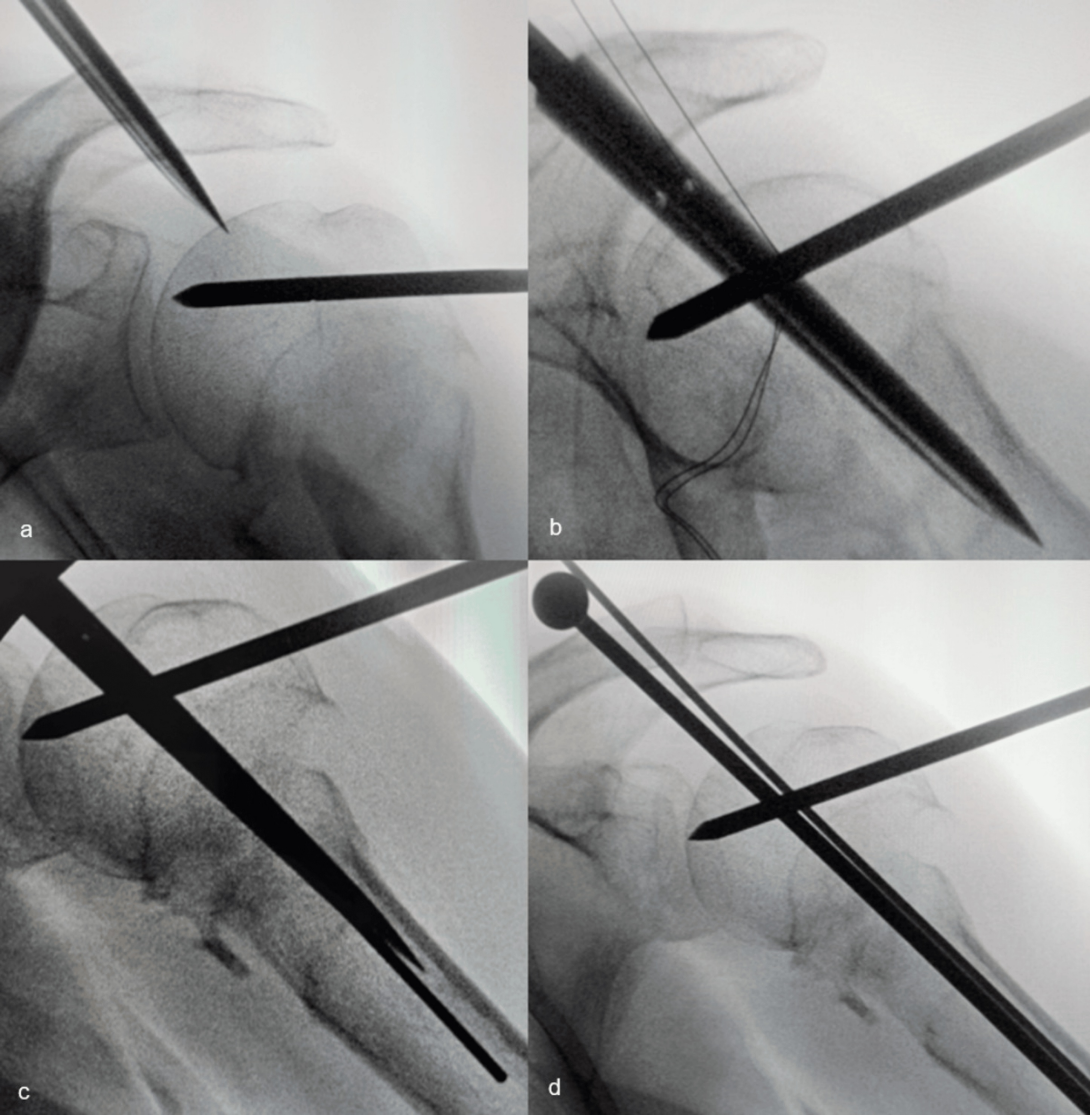
Shish kebab number one: steps in introducing the modified Steinmann pin A Steinmann pin, brought in from the lateral side, is partially correcting the varus position (a), so the humeral head can be opened at its highest point with an Aequalis™ starter awl under AP (a) and lateral (b) fluoroscopy control. A guide pin is passed down the starter awl into the medullary canal (c), the starter awl is removed, and the modified Steinmann pin is introduced in the same opening next to the guide pin (d), which is then also removed.

**Figure 7:**
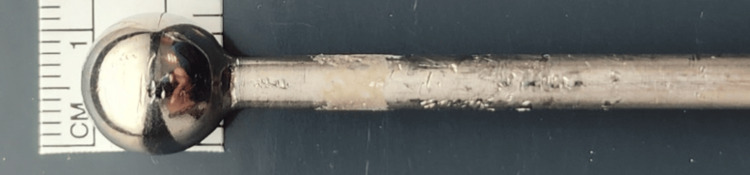
The modified Steinmann pin has a 5 mm diameter and 15 cm length, and at its proximal end, a ball with a diameter of 12 mm

**Figure 8:**
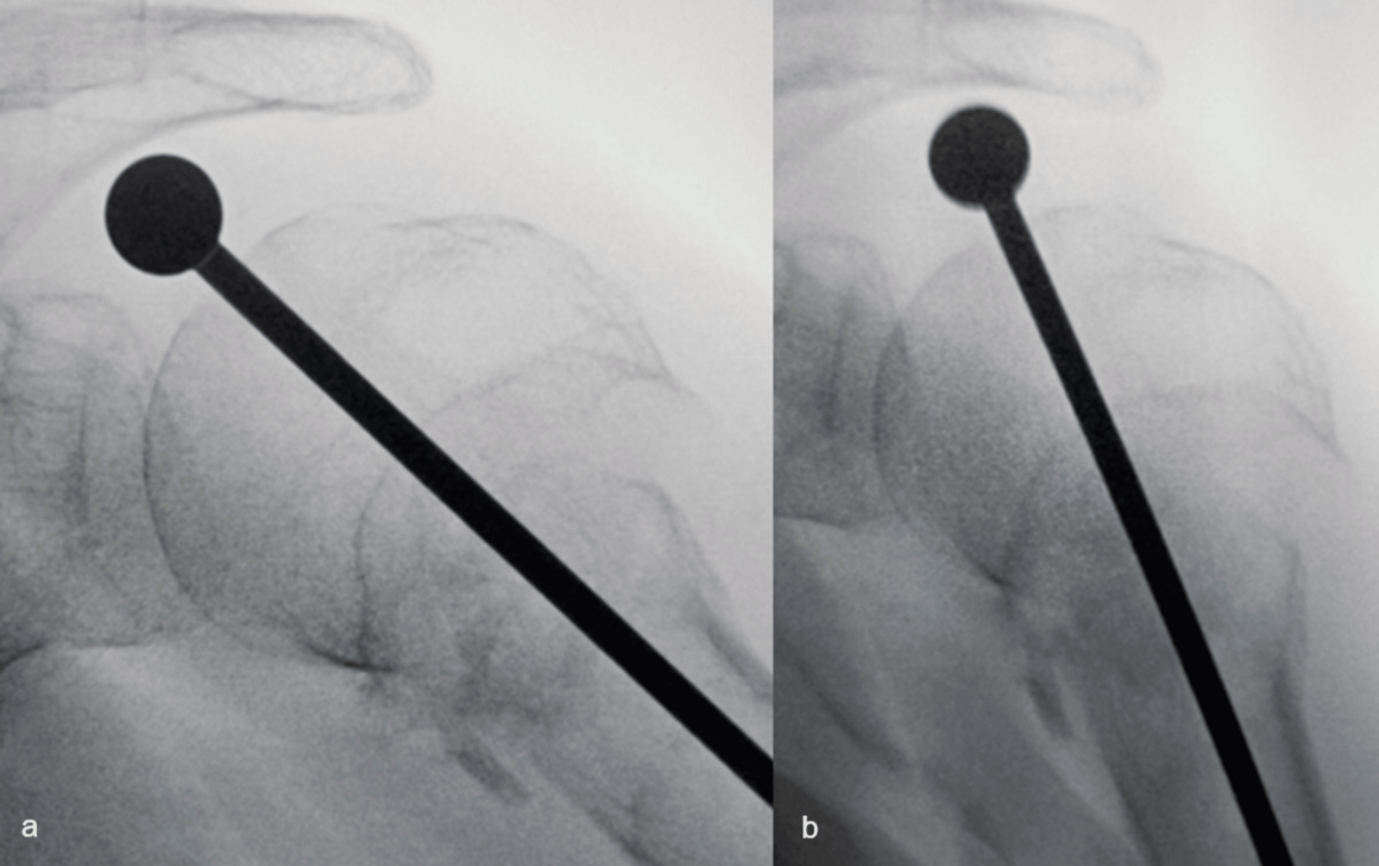
The Steinmann pin is introduced (a) and holds the reduction when the arm is lowered (b) The modified Steinmann pin is introduced until it touches the cartilage of the humeral head (a). After that, the arm is lowered to the side of the patient, keeping the humeral head in its reduced position (b). A deltopectoral approach can now be performed, during which the arm can be placed in any desired position, while the Steinmann pin will keep the head reduced.

After draping, a Steinmann pin was percutaneously placed in the humeral head to partially correct its varus position. A 1 cm incision was then used to create the Neviaser portal, through which the cannulated starter awl of the Aequalis™ IM Humeral Nail (Tornier, France) was introduced to create an opening starting at the highest point of the humeral head in both the AP and lateral projections (Figures 6a, 6b). A guide pin was then introduced through the awl so as not to lose the position of the entry hole in the humeral head on fluoroscopy (Figure 6c). Next, working through the same Neviaser portal, a modified Steinmann pin with an enlarged round head (Figure 7) was inserted next to the guide pin in the same opening in the humeral head (Figure 6d), after which the guide pin was removed, and the modified Steinmann pin was then punched in until its round head contacted the cartilage of the humeral head (Figure 8a).

With the Steinmann pin now holding the humeral head in its reduced position, the arm was lowered to the side of the patient (Figure 8b) to perform a deltopectoral approach. It was now possible to position the arm in any position needed during this approach (adducted, abducted, extended, etc.) without losing the position of the proximal humeral fragment relative to the humeral shaft.

Introducing the Steinmann pin through the highest point of the humeral head will correct any displacement in the frontal and sagittal planes. In surgical neck fractures (no tuberosity involvement), there usually is no or very limited rotational deformity in the horizontal plane. The construct with the modified Steinmann pin even has some rotational stability due to the incongruous shape of both bone ends and the fibrous tissue surrounding the non-union site. If, during the preparation of the humeral shaft, some rotational shifts do occur, then those are now easily corrected by realigning the bicipital groove of the proximal fragment with that of the distal fragment.

**Figure 9:**
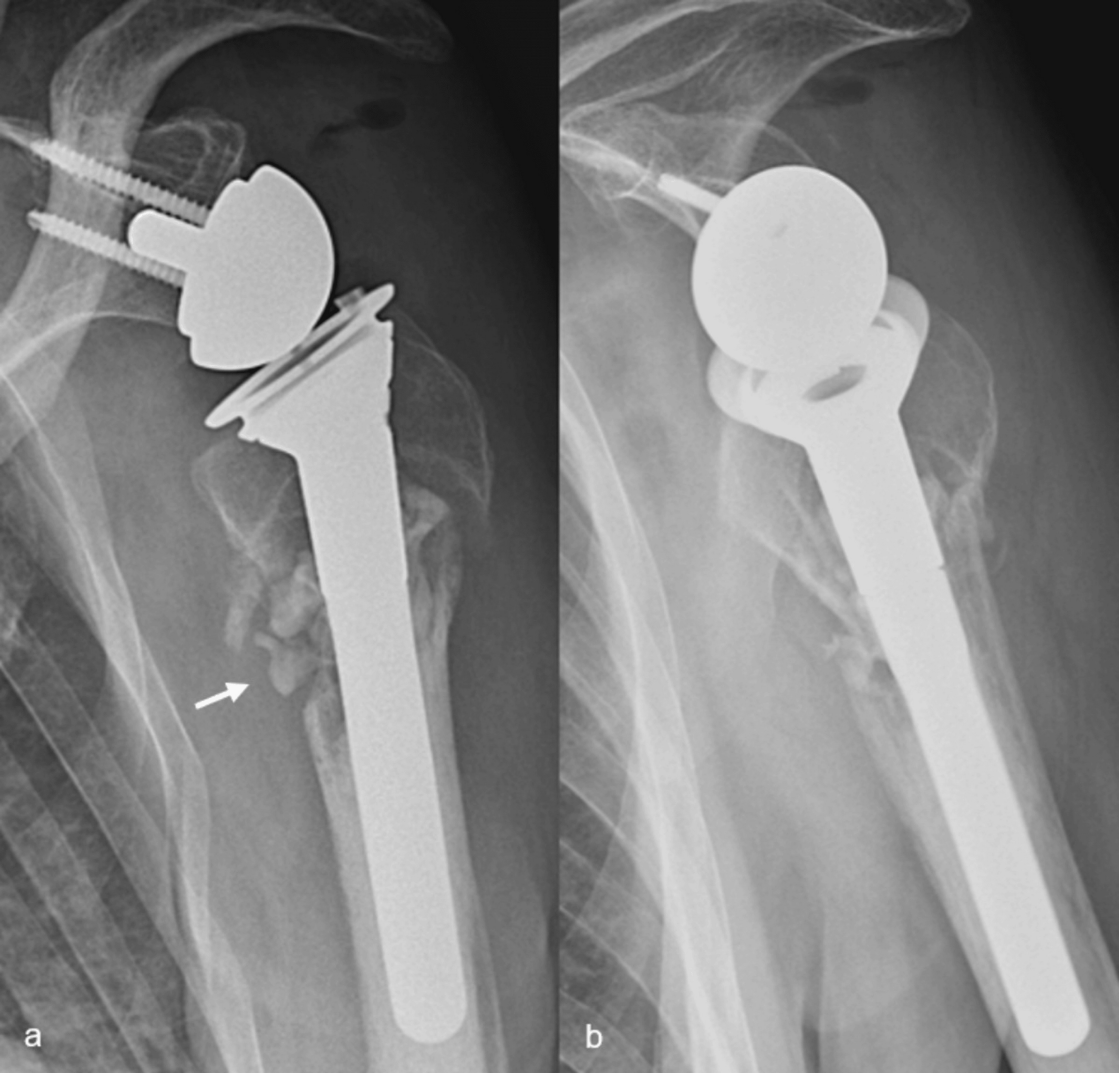
Shish kebab number two: the humeral stem is introduced through the proximal humeral fragment into the medullary canal Postoperative AP (a) and lateral (b) X-rays showing the humeral stem "shish kebabbed" through the proximal humeral fragment into the medullary canal. The distal part of the stem is cemented, while the fixation of the proximal part relies on its Trabecular Metal™ coating. There is some extrusion of cement into the non-union site (arrow).

The humeral head was then approached through a subscapularis tenotomy; the modified Steinmann pin was removed, after which the humeral and glenoid sides were prepared in the usual manner. During this part of the procedure, the position of the humeral head is held by the humeral reamers and the humeral trial component. In the end, the humeral stem was implanted with cement, completing step two of the "shish kebab technique." In an effort to limit cement extrusion at the non-union site, only the round non-coated distal part of the Trabecular Metal™ reversed humeral stem (Zimmer-Biomet, USA) was cemented, relying on the Trabecular Metal™ coating of the proximal part of the humeral stem for press-fit ingrowth in the viable proximal humeral fragment (Figure 9). The inlay design of the TM reversed humeral stem increases the bone-prosthesis interface at the metaphyseal level. This facilitates primary fixation in the metaphyseal fragment, encourages subsequent bony ingrowth of the stem, and limits the risk of bone resorption due to stress shielding [18,19].

Our postoperative protocol did not differ from our usual protocol after a standard reverse shoulder arthroplasty. After one week of strict immobilization of the operated shoulder, a home exercise program was started, consisting mainly of pendulum exercises and passive elevation, to be repeated three times a day. Three weeks after the operation, formal physiotherapy was started, including active elevation. No formal shoulder strengthening exercises were prescribed.

At the last visit, six months postoperatively, the patient was pain-free and could handle most activities of daily living. Active shoulder elevation had progressed to about 120°, with excellent rotational control. Further clinical and radiological follow-up is scheduled on a yearly basis.

## Discussion

The "two-step shish kebab technique" is a reliable and reproducible technique when reverse arthroplasty for surgical non-unions of the proximal humerus in the elderly patient is indicated. It restores the proximal humerus bone stock and secures functional outcome and stability of the prosthesis. The main advantage of adding the first "shish kebab" lies in avoiding having to struggle with the position of the proximal humeral fragment in the frontal, sagittal, and horizontal planes during the deltopectoral exposure. The first part temporarily holds the reduction, while the second allows fixation of the correctly reduced proximal humeral fragment to the rest of the humerus.

Our 'skewer' consists of a modified Steinmann pin with a ball-shaped head (Figure 7). To our knowledge, this pin is not commercially available off-the-shelf. We had several of these pins manufactured at a low cost by one of the companies that produces custom-made orthopedic tools. We use those pins on a regular basis for percutaneous reduction of the humeral head fragment during proximal humeral nailing.

Alternatives are possible, such as intramedullary reamers with a proximal end larger than the reamer size itself or pins with a self-made curved top end. The necessary specifications are, however, that the thickness of the 'pin' should be sufficient so as not to bend when the arm position is changed, and to have enough grip in the proximal humeral fragment. The size of the part sitting on top of the humeral head should be large enough not to 'fall' in the humeral intramedullary canal and at the same time small enough to easily pass through a percutaneous (Neviaser) portal and not to encroach on the acromion to avoid limited motion of the arm while performing the deltopectoral approach.

It is probably feasible to omit the part where we used the Aequalis^TM^ starter awl and guide pin and to start directly with the modified Steinmann pin, provided it can be held firmly enough to be able to open the humeral head and pass through the non-union site into the humeral intramedullary canal.

In the case presented, the distal part of the humeral stem was cemented. If humeral components are used that allow reliable distal fixation through distal coating and/or distal stem shape, this procedure might be done cementless.

Also, in this case, the amount of varus of the humeral head lends itself to introducing the Steinmann pin via a Neviaser portal. In instances where the head position is more neutral or in valgus, a more classic portal can be used directly anterior to the AC joint.

It is important to restore humeral length and proximal bone stock as much as possible to maximize active function and stability, preserving the deltoid wrapping effect. In our case, there was an excavation of the humeral head, but a very limited loss of cortical bone on both sides of the non-union. However, preoperatively, we had bilateral X-rays made with length measurements of both humeri to assess any significant bone loss. Perioperatively, the upper border of the pectoralis major serves as an important reference point for the correct insertion height of the humeral component [20]. Stability testing with the trial components in place should, of course, not be omitted either.

## Conclusions

The "two-step shish kebab technique" offers a practical and effective solution for managing surgical non-unions of the proximal humerus using reverse shoulder arthroplasty in elderly patients. By temporarily securing the proximal humeral fragment prior to exposure of the fracture site and facilitating controlled, accurate fixation, it restores the proximal humerus bone stock and secures functional outcome and stability of the prosthesis. This technique enhances intraoperative handling and might improve postoperative outcomes in these specific cases. These conclusions, however, are based on a single case and require further research involving comparative studies, large patient cohorts, and long-term follow-up to validate these findings as well as the efficacy and safety of the described technique in surgical neck non-unions of the proximal humerus.
